# Pulmonary Artery Aneurysm Associated With Aspergilloma in a Patient Diagnosed With Granulomatosis With Polyangiitis

**DOI:** 10.7759/cureus.41132

**Published:** 2023-06-29

**Authors:** Ashbina Pokharel, Indira Acharya, Joseph Skender

**Affiliations:** 1 Internal Medicine, Beaumont Hospital, Royal Oak, USA; 2 Internal Medicine, MedStar Union Memorial Hospital, Baltimore, USA; 3 Rheumatology, Beaumont Hospital, Royal Oak, USA

**Keywords:** anca-associated vasculitis, aneurysm, massive hemoptysis, aspergilloma, granulomatosis with polyangiitis (gpa)

## Abstract

Granulomatosis with polyangiitis (GPA) is an autoimmune, necrotizing granulomatous disease that affects small- and medium-sized blood vessels. Aspergilloma is a fungal mass of Aspergillus and usually found in the preexisting cavity in lung parenchyma. Surgical resection is the mainstay of treatment of aspergilloma. In this article, we present a case of a 70-year-old male with GPA and aspergilloma who presented with massive, life-threatening hemoptysis. Further workup with a chest computed tomography (CT) pulmonary angiogram demonstrated a pulmonary artery pseudoaneurysm along the wall of the lung cavity which was emergently managed with embolization and required monitoring in the medical intensive care unit. This case report alerts clinicians to maintain a high level of suspicion for an aneurysm if the degree of hemoptysis is higher than expected.

## Introduction

Granulomatosis with polyangiitis (GPA) is a rare systemic autoimmune vasculitis with positive antineutrophil cytoplasmic antibodies (ANCA). GPA involves a triad of upper respiratory symptoms (sinusitis, saddle nose, mastoiditis), lower respiratory tract symptoms (lung nodules, alveolar hemorrhage), systemic vasculitis, and kidney involvement [1]. Treatment depends on the extent of clinical severity and usually involves immunosuppressives. Aspergilloma is a fungus ball of Aspergillus hyphae that thrives in poorly drained and avascular cavitary spaces. It is common in immunocompromised patients and immunocompetent patients with preexisting lung disease [2]. Management of aspergilloma includes surgical resection, antifungal agents, and arterial embolization in cases of massive hemoptysis. Not only is it difficult to diagnose, but also a high degree of suspicion should be on the clinician's mind regarding the complications of both GPA and aspergilloma when these clinical entities coexist, and this is a challenge to manage. The objective of this article is to present a challenging diagnostic case in which GPA and aspergilloma coexist and to discuss the management of life-threatening complications.

## Case presentation

A 70-year-old man with a past medical history of diabetes mellitus, hypertension, and GPA on prednisone presented to the emergency department with hemoptysis 21 days before presentation. He was diagnosed with GPA 20 years ago and was treated with rituximab and corticosteroids in the past. He was in remission for the past three years. He started having a dry cough and chest pain six months ago. A chest CT revealed an 8 cm central perihilar cavitary mass in the superior aspect of the right lower lobe. Additional chest CT findings included band-like scarring adjacent to the mass, a 3 mm right upper lobe pulmonary nodule, a 6 mm left upper lobe pulmonary nodule, and a band-like area of scarring involving the superior aspect of the left major fissure. Bronchoscopy with bronchoalveolar lavage confirmed Aspergillus. He was treated with voriconazole for one month which improved his cough and shortness of breath which further supported the diagnosis of aspergilloma. Twenty-one days prior to the presentation, he started having intermittent hemoptysis along with cough. So, he was started on voriconazole 200 mg twice daily, and the dose of prednisone was decreased from 20 mg to 10 mg daily. The cough improved with voriconazole, and the hemoptysis continued and gradually worsened. Prior to arriving at our center, he coughed up one cup of fresh blood along with clots. Additionally, he reported chest pain that worsened with inspiration and lying on the right side.

**Figure 1:**
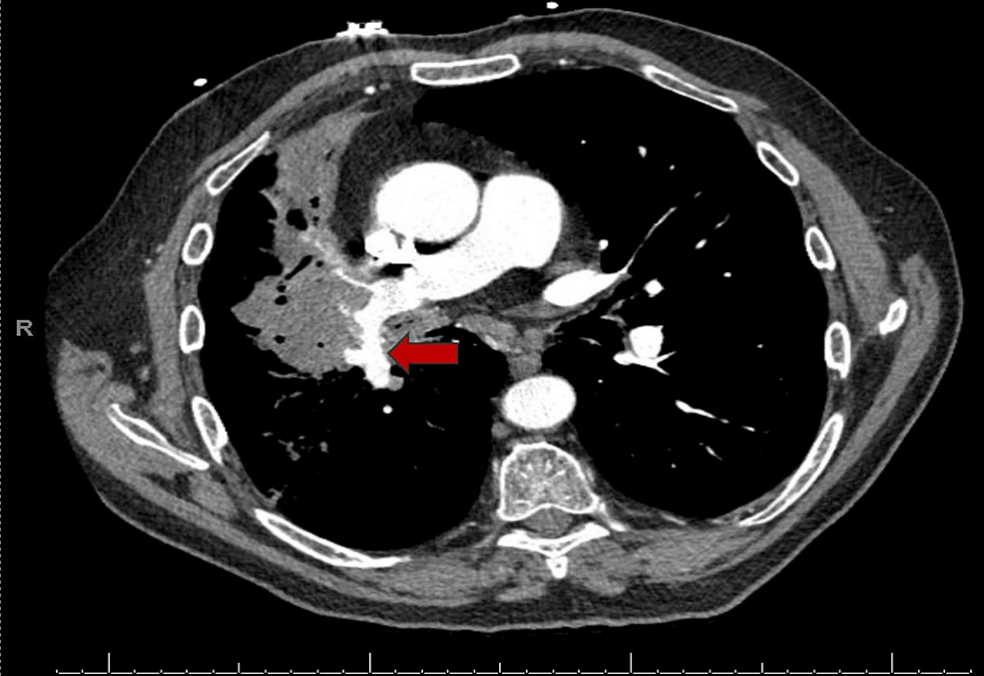
CT pulmonary angiogram demonstrating pseudoaneurysm of the right interlobar pulmonary artery (red arrow)

**Figure 2:**
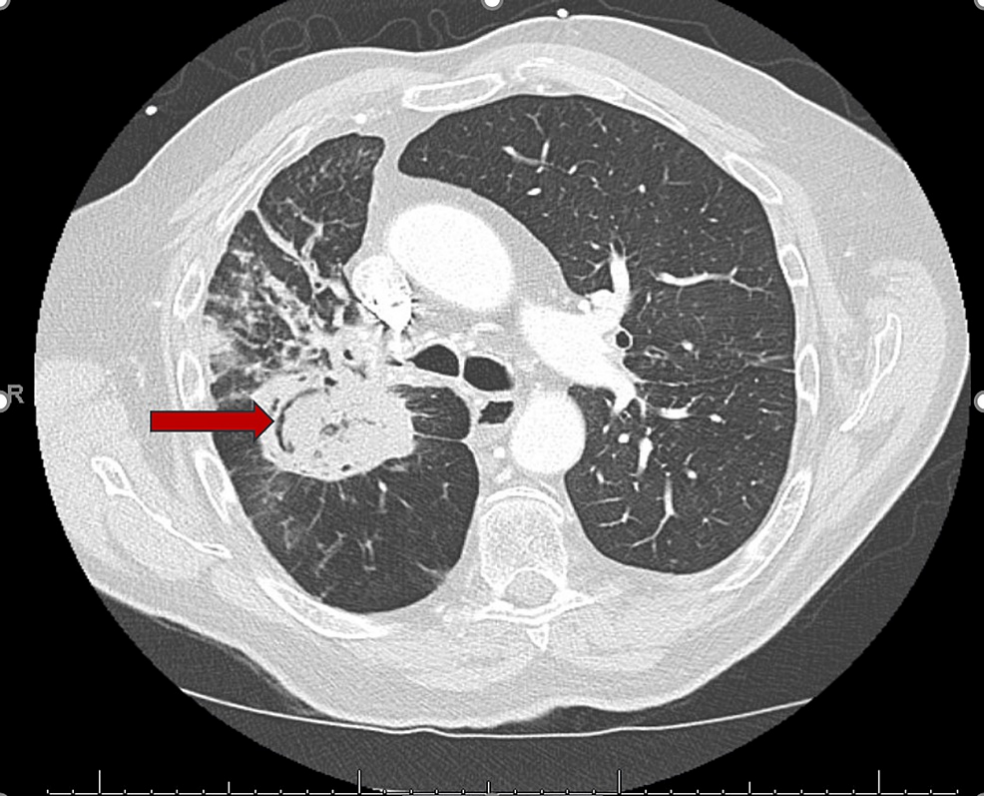
Chest CT scan demonstrating 6 cm aspergilloma (red arrow)

Vital signs on admission were blood pressure of 111/ 74 mm hg, heart rate of 105/ min, respiratory rate of 22/ min, and oxygen saturation of 97% on five liters of oxygen. On physical examination, he was coughing with hemoptysis, and mild expiratory wheezing was heard bilaterally on lung bases. Lab findings showed hemoglobin of 10.3 g/dL (reference range: 13.5 - 17 g/dL), compared to 11.5 g/dL a month prior. The remaining pertinent laboratory results are shown in Table 1. A CT pulmonary angiogram of the chest revealed a 12 mm pseudoaneurysm in the right interlobar pulmonary artery (Figure 1) along the wall of the right central cavitary mass (Figure 2). He was evaluated by the surgical team. Given the central location of aspergilloma, management will require pneumectomy which is aggressive compared to embolization. So, after a multidisciplinary team discussion with the patient, we decided to move ahead with embolization.

**Table 1:** Lab findings on presentation ANCA: Antineutrophil cytoplasmic antibody

Labs	Results	Reference range
White blood cell count	20.5 bil/L	3.5 - 10.1 bil/L
Hemoglobin	10.3 g/dL	13.5 - 17 g/dL
Platelets	315 bil/L	150 - 400 bil/L
Protime	13.1 seconds	9.2 - 13.5 seconds
Sodium	138 mmol/L	135 - 145 mmol/L
Potassium	3.9 mmol/L	3.5 - 5.2 mmol/ L
Creatinine	0.84 mg/dL	0.60 - 1.30 mg/dL
ANCA titer	< 1:20	< 1: 20

Subsequently, he underwent a catheter angiogram with coil embolization of the pseudoaneurysm. Postembolization, he remained intubated and was managed in the medical intensive care unit. He continued to remain hemodynamically stable and minimal bloody output was observed in the endotracheal tube for two days. On the third day, he underwent bronchoscopy, and no bleeding was noted. As a result, he was extubated after the bronchoscopy and closely monitored in the ICU for two more days. His hemoptysis significantly improved, and his hemoglobin remained stable. He was discharged with continuation of voriconazole and prednisone 10 mg daily. He was recommended to follow up with the surgical team after discharge for further management of aspergilloma.

## Discussion

GPA is a rare necrotizing granulomatous vasculitis that affects small- to medium-sized vessels. It commonly involves the upper respiratory tract, lungs, and kidneys, with isolated sinonasal disease present in 25% of cases [3]. GPA belongs to a group of ANCA vasculitis, which also includes eosinophilic granulomatosis with polyangiitis and microscopic polyangiitis [4]. Although the exact cause of GPA is not known, a complex interaction involving genetics, environmental factors, and microbiomes has been implicated in the pathogenesis, with ANCA presumed to be responsible for inflammation [1,4]. The diagnosis can be made according to the American College of Rheumatology criteria or the ELK (E stands for ear, nose, and throat or upper respiratory tract, L for lung, and K for kidney) criteria [1]. Treatment includes immunosuppressive agents, among which commonly used agents are cyclophosphamide, glucocorticoids, rituximab, azathioprine, methotrexate, and plasmapheresis if needed. Cyclophosphamide with glucocorticoids has been effective in severe disease, while recent data have favored rituximab as a preferable option for both induction and maintenance of remission [1,5].

Aspergilloma (a noninvasive type of chronic pulmonary aspergillosis) is a mycetoma composed of Aspergillus hyphae combined with cellular debris and mucus [2]. The most common risk factors for Aspergillus infection include cavitary lung disease, chronic debilitating conditions, and an immunosuppressed state. In our case, the contributing risk factors were pulmonary disease secondary to GPA and immunosuppression due to chronic steroid use. Hemoptysis is the most common clinical manifestation in symptomatic patients and was also present in our case.

Diagnosis is made based on clinical findings and characteristic imaging, combined with microbiological culture or serological evidence. Chest CT findings in aspergilloma are characterized by a mass with soft tissue attenuation within a lung cavity [6]. In our case, the diagnosis was established with CT scan findings, clinical manifestations (cough, shortness of breath), and bronchoalveolar lavage culture of Aspergillus. Though oral triazoles (voriconazole, itraconazole) are the standard treatment for chronic cavitary pulmonary aspergillosis [7], surgical resection is the preferred management option for aspergilloma [2]. Minimally symptomatic patients can be managed with antifungals [2]. In our patient, voriconazole initially improved his hemoptysis. However, retrospective clinical analysis alerted us to the fact that earlier surgical management when hemoptysis was first noted could have altered the severity and course of hemoptysis and consequently the management approach. Though surgical resection is standard management for aspergilloma, our patient was managed with embolization rather than surgery. In our case, involvement of aspergilloma was in the central lung and would require pneumectomy which is aggressive compared to embolization. So, after multidisciplinary team discussion with the patient, we agreed to proceed with embolization.

## Conclusions

We present a rare case of both GPA and aspergilloma in the same individual with life-threatening hemoptysis. This case emphasizes the importance of early management of aspergilloma with new hemoptysis, regardless of its severity. Close clinical monitoring and subsequent imaging to evaluate the vascular source of hemoptysis should be at the forefront of the clinician's considerations when clinical symptoms are concerning.
